# Central corneal thickness and its associations in a Russian population. The Ural eye and Medical Study

**DOI:** 10.1038/s41433-022-02026-1

**Published:** 2022-03-28

**Authors:** Mukharram M. Bikbov, Timur R. Gilmanshin, Rinat M. Zainullin, Gyulli M. Kazakbaeva, Artur F. Zaynetdinov, Ildar F. Nuriev, Songhomitra Panda-Jonas, Inga I. Arslangareeva, Ainur A. Zinnatullin, Dilya F. Yakupova, Ellina M. Rakhimova, Yulia V. Uzianbaeva, Renat I. Khikmatullin, Nikolay A. Nikitin, Said K. Aminev, Svetlana R. Mukhamadieva, Venera F. Mavlieva, Jost B. Jonas

**Affiliations:** 1 Ufa Eye Research Institute, Ufa, Bashkortostan Russia; 2 Institute of Clinical and Scientific Ophthalmology and Acupuncture Jonas & Panda, Heidelberg, Germany; 3 Department of Ophthalmology, Medical Faculty Mannheim of the Ruprecht-Karls-University of Heidelberg, Mannheim, Germany

**Keywords:** Visual system, Optic nerve diseases

## Abstract

**Background:**

To assess central corneal thickness (CCT) and its associations in a Russian population.

**Methods:**

The population-based Ural Eye and Medical Study included 5899 (80.5%) out of 7328 eligible individuals. As part of an ophthalmological and general examination, CCT was measured by Scheimflug imaging.

**Results:**

The study included 5792 (98.2%) participants (age:58.8 ± 10.6 years;range: 40–94 years) with available bilateral CCT measurements. Mean CCT was larger in Russians than non-Russians (549.5 ± 32.8 µm versus 539.2 ± 33.9 µm; *P* < 0.001). In multivariable analysis, thicker CCT was associated (regression coefficient r:0.43) with younger age (standardized regression coefficient beta:−0.09; non-standardized regression coefficient B:−0.29;95% confidence interval (CI):−0.39,−0.20; *P* < 0.001), male sex (beta:0.05; B:3.10; 95%CI:1.18,5.03; *P* = 0.002), urban region of habitation (beta:0.10; B:6.83; 95%CI:4.61, 9.05; *P* < 0.001), Russian ethnicity (beta:0.04; B:3.48; 95%CI:1.04, 5.91; *P* = 0.005), higher level of education (beta:0.04; B:0.97;95%CI:0.29,1.66; *P* = 0.006), higher serum bilirubin concentration (beta:0.05;B:0.15; 95%CI:0.07,0.23;*P* < 0.001), lower corneal refractive power (beta:−0.09;B:11.92; 95%CI:−2.50,−1.35; *P* < 0.001), smaller anterior chamber angle (beta:−0.07;B:−0.38;95%CI:−0.52,−0.24;*P* < 0.001), higher IOP readings (beta:0.38; B:3.47; 95%CI:3.21,3.73; *P* < 0.001), and higher rise in IOP readings by medical mydriasis (beta:0.07; B:0.88;95%CI:0.54,1.22;*P* < 0.001). In that model, CCT was not associated with body height (*P* = 0.14), previous cataract surgery (*P* = 0.10), axial length (*P* = 0.18) or prevalence of glaucoma (*P* = 0.11). The mean inter-eye difference in CCT was 8.52 ± 13.9 µm (median:6.0;95CI:8.16,8.88). A higher inter-eye CCT difference was associated with older age (beta:0.08; B:0.11;95%CI:0.07,0.15; *P* = 0.01), lower level of education (beta:−0.04;B:−0.34; 95%CI:−0.60,−0.08; *P* < 0.001) and status after cataract surgery (beta:0.04; B:2.92;95%CI:1.02,4.83; *P* = 0.003).

**Introduction conclusions:**

In this ethnically mixed population from Russia with an age of 40+ years, mean CCT (541.7 ± 33.7 µm) was associated with parameters such as younger age, male sex, Russian ethnicity, and higher educational level. These associations may be taken into account when the dependence of IOP readings on CCT are considered. Glaucoma prevalence was unrelated to CCT.

## Introduction

Central corneal thickness (CCT) is a clinically important parameter in the diagnosis of glaucoma, since the measurement of intraocular pressure (IOP) markedly depends on CCT [1–5]. It has additionally been discussed that a thin cornea may be a structural risk factor for an increased susceptibility for glaucomatous optic nerve damage at a given IOP [6, 7]. It has therefore become clinical routine to measure CCT to correct the IOP readings for their dependence on CCT. In previous hospital-based studies and population-based investigations, CCT has been measured in various ethnic populations such as Western Europeans, East Asians including Japanese and Chinese, Mongolians, Malay, Indians and Hispanics [8–15]. None of these studies however assessed the CCT in a population from Russia, and in particular did not take into account the multi-ethnicity of the total population of Russia. In addition, in most of the previous investigations, associations of CCT with other parameters were tested for an only relatively small number of variables. We therefore conducted this study to measure the CCT in an ethnically mixed population Russia and to assess associations of CCT with a large number of other ocular parameters and systemic and medical variables.

## Methods

The Ural Eye and Medical Study is a population-based investigation which was performed in the Russian republic of Bashkortostan at the southwestern end of the Ural Mountains in the study period from 2015 to 2017 [16, 17]. Study regions were Ufa as capital of Bashkortostan in a distance of about 1400 km East of Moscow and a rural region in the Karmaskalinsky District in a distance of 65 km from Ufa. The republic of Bashkortostan located between the Volga River and the Ural Mountains, is with a population of 4 million people the most populous republic in Russia. Inclusion criteria for the study were living in the study regions and an age of 40 years or older. The Ethics Committee of the Academic Council of the Ufa Eye Research Institute approved the study design and confirmed that the study adhered to the Declaration of Helsinki, and all participants gave an informed written consent. Out of a total group of 7328 eligible individuals, 5899 (80.5%) individuals (3319 [56.3%] women) with a mean age of 59.0 ± 10.7 years (range: 40–94 years) participated in the study. The study population did not differ significantly in the gender and age distribution from the Russian population as explored in the census carried out in 2010 [16].

Using a bus, the study participants were brought from their homes to the Ufa Eye Institute where a team of about 20 trained social workers, technicians and ophthalmologists performed all examinations. As also described in detail previously, the series of examinations started with a detailed interview consisting of more than 250 standardized questions on the socioeconomic background, smoking and alcohol consumption, physical activity, diet, depression and anxiety, and known diagnosis and therapy of major diseases [17, 18]. The examinations further included anthropometry, blood pressure measurement, handgrip dynamometry, spirometry, and biochemical analysis of blood samples taken under fasting conditions. We defined arterial hypertension according to the new criteria published by the American Heart Association, and criteria for the diagnosis of diabetes mellitus were a fasting serum glucose concentration of ≥7.0 mmol/L or a self-reported history of physician-based diagnosis or therapy of diabetes mellitus.

The series of ophthalmologic examinations consisted of measurement of visual acuity including automated and subjective refractometry (Auto-2Ref/Keratometer HRK-7000A HUVITZ Co, Ltd., Gyeonggi-do, Korea), perimetry (PTS 1000 Perimeter, Optopol Technology Co., Zawercie, Poland), Scheimflug imaging of the anterior segment, slit lamp-based biomicroscopy of the anterior and posterior ocular segment, non-contact tonometry (Tonometer Kowa KT-800, Kowa Company Ltd., Hamamatsu City, Japan), re-assessment of the anterior segment and lens for the presence of pseudoexfoliation after medical mydriasis, photography of the cornea and lens (Topcon slit lamp and camera, Topcon Corp. Tokyo, Japan), optical coherence tomography (OCT) (RS-3000, NIDEK co., Ltd., Aichi Japan) of the peripapillary retinal nerve fibre layer, optic nerve head and macula, and assessment of the degree of fundus tessellation using the fundus photographs. Using an anterior segment imaging device (Pentacam HR, Typ70900, OCULUS, Optikgeräte GmbH Co., Wetzlar, Germany), we measured the CCT by Scheimflug imaging. We applied the automatic mode for the data assessment. In the case of CCT readings out of the range of expectation, the measurements were repeated. All measurements were performed by the same ophthalmologist trained and supervised in the technique. Nuclear lens opacities were differentiated into 6 grades using the classifying scheme for cataract of the Age-Related Eye Disease Study [19]. We defined the presence of nuclear cataract as a nuclear cataract grade of 3 or higher. Cortical lens opacities and posterior subcapsular opacities were graded using photographs taken by retro-illumination (Topcon slit lamp and camera, Topcon Corp. Tokyo, Japan). Using a grid, we measured the percentage area of opacity. Age-related macular degeneration (AMD) was defined as suggested by the recent Beckman Initiative for Macular Research Classification Committee [20]. Glaucoma was defined by morphological criteria as described by Foster and colleagues [21].

Inclusion criterion for the present study was the availability of CCT measurements. The data of only one randomly selected eye per individual was taken for statistical analysis. The data were statistically analyzed using a statistical software package (SPSS for Windows, version 25.0, IBM-SPSS, Chicago, IL, USA). We assessed the mean values of the parameters (expressed as mean and standard deviation or as mean and 95% confidence intervals (CI)) and examined associations between CCT and other systemic parameters and ocular parameters, first in a univariable analysis, followed by a multivariable analysis. The latter included the CCT as dependent variable and as independent parameters all those variables that were associated (*P* < 0.05) with CCT in the univariable analyses. Out of the list of independent variables, we then dropped parameters due to collinearity with other independent variables or if they were no longer significantly associated with CCT. We calculated odds ratios (OR) and their 95% CI. All *P*-values were two-sided and considered statistically significant when the values were less than 0.05.

## Results

Out of 5889 participants of the Ural Eye and Medical Study, the present study included 5792 (98.2%) individuals (mean age: 58.8 ± 10.6 years; range: 40–94 years) with available bilateral CCT measurements. The study population consisted of 1170 (20.2%) Russians, 1045 (18.0%) Bashkirs, 2394 (41.3%) Tartars, 579 (10.0%) Chuvash, 21 (0.4%) Mari, and 583 (10.1%) other or undefined ethnic groups. The group of individuals with CCT measurements and participating in the present investigations as compared to the group of those without CCT readings was significantly younger (58.8 ± 10.6 years versus 67.5 ± 11.4 years; *P* < 0.001) and included proportionally more women (*P* = 0.03).

**Fig. 1:**
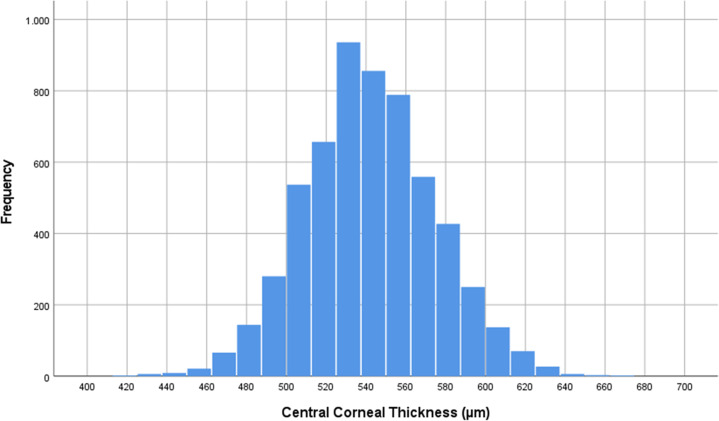
Central Corneal Thickness. Histogram showing the distribution of central corneal thickness in the Ural Eye and Medical Study.

**Fig. 2:**
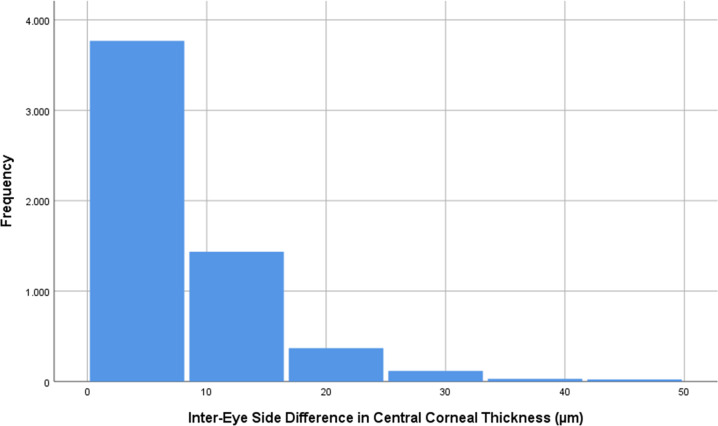
Bilateral Difference in Central Corneal Thickness. Histogram showing the distribution of the inter-eye side difference in central corneal thickness in the Ural Eye and Medical Study.

Mean CCT was 541.7 ± 33.7 µm in the right eyes (median: 541 µm; range: 200–779 µm) and 543.1 ± 33.7 µm in the left eyes (median: 543 µm,; range: 174–801 µm) with a significant (*P* < 0.001) difference between both eyes (Figs. 1,2). The mean CCT was 549.5 ± 32.8 µm (median: 548 µm; range: 397–664 µm) in the Russian group and it was 539.2 ± 33.9 µm (median: 538 µm; range: 200–779 µm) in the non-Russian group, with a significant (*P* < 0.001) difference between both groups.

In univariable analysis, thicker CCT was associated (*P* < 0.05) with the systemic parameters of older age, male sex, urban region of habitation, Russian ethnicity, taller body height, heavier body weight, higher body mass index, higher socioeconomic score and higher level of education, higher prevalence of current smoking and higher number of cigarette smoking package years, higher prevalence if any alcohol consumption, higher prevalence of a history of unconsciousness, menopause and diabetes mellitus, higher serum concentration of alanine aminotransferase, aspartate aminotransferase and total bilirubin and haemoglobin, lower serum concentration of urea, lower erythrocyte sedimentation rate, higher erythrocyte count, higher percentage of segment nuclear granulocytes on total leucocytes, higher estimated glomerular filtration rate and lower prevalence of chronic kidney disease and anaemia, higher prevalence of diabetes mellitus, lower hearing loss score, and higher dynamometric hand grip force (Table 1). Thicker CCT was associated with the ocular parameters of longer axial length, lower corneal refractive power, higher corneal volume, lower anterior chamber volume and smaller anterior chamber angle degree, lower degree and prevalence of cortical cataract, higher IOP readings before and after medical mydriasis, lower difference between the IOP readings obtained before and after medical inducing mydriasis, thicker retinal thickness measured 300 µm temporal to the fovea, higher prevalence of angle-closure glaucoma and diabetic retinopathy, higher Schirmer test, and lower prevalence of reticular pseudodrusen of the macula (Table 2).

**Table 1:** Associations (univariable analysis) between central corneal thickness systemic parameters in the Ural Eye and Medical Study.

Parameter	Interval	Standardized regression coefficient beta	Non- standardized regression coefficient B	95% confidence interval of B	*P*-Value
Age	1-year intervals	−0.09	−0.27	−0.35, −0.19	<0.001
Gender	Men / Women	−0.06	−3.72	−5.47, −1.97	<0.001
Region of habitation	Rural / Urban	0.14	9.47	7.72, 11.2	<0.001
Ethnicity	Any other ethnicity / Russian	0.12	10.1	7.91, 12.2	<0.001
Body height	1 cm	0.07	0.27	0.17, 0.37	<0.001
Body weight	kg	0.08	0.19	0.13, 0.25	<0.001
Body mass index	kg/m^2^	0.05	0.31	0.14, 0.49	<0.001
Waist circumference	cm	0.03	0.06	−0.001, 0.13	0.053
Hip circumference	cm	0.01	0.03	−0.04, 0.10	0.44
Waist/hip circumference ratio	Ratio	0.03	9.21	−0.39, 18.8	0.06
Socioeconomic Score	Score	0.09	2.00	1.43, 2.57	<0.001
Level of education	Illiteracy / Passing 5^th^ Grade / 8th Grade / 10th Grade / 11th Grade / Graduates / Specialized Secondary Education / Post Graduates	0.10	2.36	1.76, 2.96	<0.001
Physical activity Score	Score	0.01	0.03	−0.09, 0.14	0.64
Smoking, currently	No / Yes	0.04	4.72	1.65, 6.89	0.001
Smoking, package years	Number	0.03	0.08	0.01, 0.15	0.03
Alcohol consumption, any	No / Yes	0.03	2.17	0.05, 4.29	0.045
In a week how many days do you eat fruits?	Number of days	0.01	0.09	−0.35, 0.53	0.67
In a week how many days do you eat vegetables?	Number of days	−0.01	−0.25	−0.86, 0.37	0.43
How much salt do you consume every day?	g	0.01	0.15	−0.24, 0.54	0.44
History of cardiovascular disorders including stroke	No / Yes	0.02	1.37	−0.68, 3.42	0.19
History of angina pectoris	No / Yes	0.01	1.11	−1.90, 4.13	0.47
History of asthma	No / Yes	0.01	2.73	−2.56, 8.03	0.31
History of arthritis	No / Yes	−0.02	−1.38	−3.32, 0.57	0.17
History of previous bone fractures	No / Yes	0.02	1.60	−0.38, 3.58	0.11
History of low back pain	No / Yes	0.02	1.34	−0.49, 3.17	0.15
History of thoracic spine pain	No / Yes	−0.004	0.32	−2.47, 1.82	0.77
History of neck pain	No / Yes	0.02	1.59	−0.41, 3.60	0.12
History of headache	No / Yes	−0.01	−0.91	−2.74, 0.91	0.33
History of cancer	No / Yes	0.01	2.47	−2.68, 7.62	0.35
History of dementia	No / Yes	−0.02	7.73	−19.0, 3.55	0.18
History of diarrhoea	No / Yes	−0.001	−0.35	−13.2, 12.5	0.96
History of iron-deficiency anaemia	No / Yes	0.01	1.29	−2.65, 5.23	0.52
History of low blood pressure and hospital admittance	No / Yes	0.01	1.97	−2.72, 6.67	0.41
History of osteoarthritis	No / Yes	0.00	0.04	−2.31, 2.40	0.97
History of skin disease	No / Yes	0.02	2.69	−1.37, 6.74	0.19
History of thyreopathy	No / Yes	0.004	0.41	−2.45, 3.26	0.78
History of tumbling	No / Yes	0.02	1.72	−0.51, 3.95	0.13
History of unconsciousness	No / Yes	0.03	3.20	0.04, 6.36	0.047
Age of the last menstrual bleeding	Years	0.02	0.15	−0.12, 0.42	0.28
Age of last regular menstrual bleeding	Years	0.03	0.17	−0.10, 0.45	0.22
History of menopause	No / Yes	−0.05	−4.52	−7.60, 1.45	0.004
History of diabetes mellitus	No / Yes	0.04	4.36	1.24, 7.49	0.006
Serum concentration of:					
Alanine aminotransferase	IU/L	0.04	0.10	0.03, 0.18	0.005
Aspartate aminotransferase	IU/L	0.03	0.10	0.02, 0.18	0.01
Aspartate aminotransferase-to- Alanine aminotransferase ratio	Ratio	−0.003	−0.19	−1.97, 1.58	0.83
Bilirubin, total	µmol/L	0.04	0.11	0.04, 0.19	0.004
High-density lipoproteins	mmol/L	−0.01	−0.52	−1.54, 0.51	0.32
Low-density lipoproteins	mmol/L	−0.003	−0.10	−0.87, 0.67	0.80
Cholesterol	mmol/L	0.01	0.24	−0.28, 0.75	0.36
Triglycerides	mmol/L	0.03	1.17	−0.06, 2.39	0.06
Rheumatoid factor	IU/mL	−0.02	−0.60	−1.54, 0.34	0.21
Erythrocyte sedimentation rate	Mm/min	−0.6	−0.19	−0.27, −0.11	<0.001
Glucose	mmol/L	0,02	0.33	−0.19, 0.85	0.22
Urea	mmol/L	−0.04	−0.90	−1.49, −0.30	0.003
Creatinine	µmol/L	0.003	0.005	−0.03, 0.04	0.79
Haemoglobin	g/L	0.07	0.15	0.09, 0.21	<0.001
Erythrocyte count	10^6^ cells / µL	0.06	5.52	3.24, 7.79	<0.001
Leucocyte count	10^9^ cells / L	0.01	0.14	−0.47, 0.75	0.65
Rod-core granulocytes	% of leucocytes	−0.01	−0.17	−0.80, 0.47	0.61
Segment nuclear granulocyte	% of leucocytes	0.03	0.15	0.03, 0.27	0.01
Eosinophil granulocytes	% of leucocytes	−0.01	−0.39	−1.25, 0.47	0.37
Lymphocytes	% of leucocytes	−0.01	−0.06	−0.20, 0.08	0.40
Monocytes	% of leucocytes	−0.04	−0.51	−0.89, −0.13	0.009
Diabetes mellitus, prevalence	Yes/No	0.04	4.32	1.59, 7.04	0.002
Estimated glomerular filtration rate	30 mL/min/1.73 m²	0.05	0.09	0.04, 0.13	<0.001
Stage of chronic kidney disease	0–5	−0.04	−0.16	−0.28, −0.04	0.007
Anaemia	No / Yes	−0.04	−3.24	−5.29, −1.19	0.002
Blood pressure, systolic	mm Hg	0.01	0.01	−0.03, 0.05	0.69
Blood pressure, diastolic	mm Hg	0.02	0.08	−0.04, 0.19	0.18
Blood pressure, mean	mm Hg	0.02	0.05	−0.02, 0.12	0.15
Arterial hypertension	Yes/No	0.01	0.76	−0.98, 2.50	0.39
Arterial hypertension, stage	0–4	0.01	0.22	−0.62, 1–07	0.61
Ankle-brachial pressure index, right		0.002	0.43	−5.91, 6.77	0.89
Prevalence of chronic obstructive pulmonary disease	Yes/No	0.01	1.19	−2.42, 4.79	0.52
Hearing loss	Hearing loss score (0–44)	−0.05	−0.15	−0.23, −0.06	0.001
Depression Score	Depression score unit (range: −4 to +15)	−0.02	−0.13	−0.36, 0.10	0.27
State-Trait Anxiety Inventory	State-Trait Anxiety Inventory Score (range: −7 to 13)	−0.03	−0.23	−0.48, 0.01	0.06
Manual dynamometry, right hand	dekaNewton	0.09	0.26	0.18, 0.34	<0.001
Manual dynamometry, right hand	dekaNewton	0.09	0.27	−0.19, 0.35	<0.001

**Table 2:** Associations (univariable analysis) between central corneal thickness and ocular parameters in the Ural Eye and Medical Study.

Parameter	Interval	Standardized regression coefficient beta	Non- standardized regression coefficient B	95% confidence interval of B	*P*-Value
Refractive error, spherical equivalent	Dioptres	0.02	0.25	0.14, 0.23	0.21
Refractive error, cylindrical value	Dioptres	0.02	0.75	−0.37, 1.86	0.19
Axial length	mm	0.04	1.32	0.52, 2.12	0.001
Corneal refractive power	Dioptres	−0.13	−2.62	−3.16, −2.08	<0.001
Corneal volume	mm^3^	0.73	5.99	5.84, 6.14	<0.001
Anterior chamber depth	mm	−0.01	−0.50	−2.28, 1.29	0.59
Anterior chamber volume	µL	−0.08	−0.08	−0.10, −0.05	<0.001
Anterior chamber angle	Degree	−0.07	−0.35	−0.48, −0.23	<0.001
Lens thickness	mm	−0.01	−1.09	−3.24, 1.07	0.32
Nuclear cataract degree	Grade	−0.02	−0.71	−1.63, 0.22	0.14
Nuclear cataract, prevalence	No / Yes	−0.01	−0.80	−2.71, 1.11	0.41
Cortical cataract, degree	Percentage	−0.04	−0.13	−0.23, −0.03	0.008
Cortical cataract, prevalence	No / Yes	−0.04	−4.19	−6.97, −1.42	0.003
Subcapsular cataract, degree	Percentage	0.01	0.16	−0.22, 0.55	0.41
Subcapsular cataract, prevalence	No / Yes	0.001	0.43	−11.8, 12.6	0.95
Fundus tessellation, macula region	Grade	0.001	0.03	−1.04, 1.10	0.95
Fundus tessellation, peripapillary region	Grade	−0.006	−0.19	−1.10, 0.72	0.68
Intraocular pressure, before Mydriasis	mmHg	0.34	2.95	2.74, 3.16	<0.001
Intraocular pressure, after Mydriasis	mmHg	0.32	2.72	2.48, 2.96	<0.001
Intraocular pressure, difference “after Mydriasis” minus “before mydriasis”	mmHg	−0.04	−0.43	−0.78, −0.07	0.02
Retinal thickness (total), fovea	µm	0.01	0.01	−0.01, 0.02	0.46
Retinal thickness (total), 300 µm temporal to the fovea	µm	0.03	0.03	0.002, 0.05	0.03
Retinal thickness (total), 300 µm nasal to the fovea	µm	0.02	0.02	−0.01, 0.04	0.14
Retinal nerve fibre layer thickness, peripapillary	µm	0.03	0.05	−0.003, 0.10	0.07
Pterygium, prevalence	No / Yes	−0.03	−8.31	−15.1, 1.61	0.02
Pseudoexfoliation,	No / Yes	−0.01	−0.94	−5.74, 3.86	0.70
Glaucoma, prevalence	No / Yes	0.01	1.82	2.79, 6.43	0.44
Glaucoma stage	0–5	−0.01	−0.71	−2.64, 1.21	0.47
Open-angle glaucoma, prevalence	No / Yes	−0.01	−1.90	−7.37, 3.56	0.50
Angle-closure glaucoma, prevalence	No / Yes	0.03	10.9	2.38, 19.3	0.01
Diabetic retinopathy, prevalence	No / Yes	0.04	9.56	2.52, 16.6	0.008
Diabetic retinopathy, ETDRS grading	Scale	0.03	0.22	−0.03, 0.46	0.08
Myopic maculopathy, stage	0–4	−0.01	−0.80	−3.37, 1.78	0.55
Age-related macular degeneration, early stage, prevalence	No / Yes	−0.01	−1.56	−5.26, 2.15	0.41
Age-related macular degeneration, intermediate stage, prevalence	No / Yes	0.002	0.40	−4.10, 4.91	0.86
Age-related macular degeneration, late stage, prevalence	No / Yes	−0.01	−1.23	−7.24, 4.79	0.69
Age-related macular degeneration, reticular pseudodrusen, prevalence	No / Yes	−0.04	−6.18	−10.5, −1.87	0.005
Age-related macular degeneration, any stage, prevalence	No / Yes	−0.01	−1.41	−4.47, 1.67	0.37
Dry eye, Schirmer test	mm	0.03	0.15	0.02, 0.29	0.03
Meibomian gland dysfunction	Grade 0–4	−0.02	−0.67	−1.70, 0.36	0.20
Visual acuity, best corrected	logMAR	−0.01	−1.00	−3.30, 1.30	0.39

In the multivariable analysis, we dropped due to collinearity, the parameters of body weight (versus body mass index); variance inflation factor (VIF: 116), package years (versus current smoking, VIF: 4.47), socioeconomic index (versus level of education, VIF:7.44), stage of chronic kidney disease (versus estimated glomerular filtration rate, VIF: 5.7), prevalence of anaemia (versus serum haemoglobin concentration, VIF: 2.4), prevalence of cortical cataract (versus degree of cortical cataract, VIF: 2.2), serum concentration of alanine aminotransferase (versus serum concentration of aspartate aminotransferase, VIF: 4.6), anterior chamber volume (versus anterior chamber angle, VIF: 2.6), and IOP after mydriasis (versus IOP before mydriasis). Due to a lack of statistic al significance, we dropped body mass index (*P* = 0.38), serum concentration of aspartate aminotransferase (*P* = 0.93), hearing loss score (*P* = 0.88), erythrocyte sedimentation rate (*P* = 0.86), percentage of segment nuclear granulocytes on total leucocytes (*P* = 0.62), serum concentration of urea (*P* = 0.59) and haemoglobin (*P* = 0.46), retinal thickness 300 µm temporal to the fovea (*P* = 0.71), prevalence of diabetic retinopathy (*P* = 0.55) and reticular pseudodrusen (*P* = 0.80), body height (*P* = 0.93), history of unconsciousness (*P* = 0.41), prevalence of diabetes (*P* = 0.61), Schirmer´s test (*P* = 0.34), dynamometric hand grip force (*P* = 0.35), prevalence of angle-closure glaucoma (*P* = 0.12), consumption any alcohol (*P* = 0.33), degree of cortical cataract (*P* = 0.25), axial length (*P* = 0.29), estimated glomerular filtration rate (*P* = 0.10), and prevalence of current smoking (*P* = 0.14).

In the final model, a thicker central corneal thickness was associated (regression coefficient r: 0.43) with younger age (standardized regression coefficient beta: −0.09; non-standardized regression coefficient B: −0.29; 95% confidence interval (CI): −0.39, −0.20; *P* < 0.001), male sex (beta: 0.05; B: 3.10; 95%CI: 1.18, 5.03; *P* = 0.002), urban region of habitation (beta: 0.10; B: 6.83; 95%CI: 4.61, 9.05; *P* < 0.001), Russian ethnicity (beta: 0.04; B: 3.48; 95%CI: 1.04, 5.91; *P* = 0.005), higher level of education (beta: 0.04; B: 0.97; 95%CI: 0.29, 1.66; *P* = 0.006), higher serum bilirubin concentration (beta: 0.05; B: 0.15; 95%CI: 0.07, 0.23; *P* < 0.001), lower corneal refractive power (beta: −0.09; B: 11.92; 95%CI: −2.50, −1.35; *P* < 0.001), smaller anterior chamber angle (beta: −0.07; B: −0.38; 95%CI: −0.52, −0.24; *P* < 0.001), higher IOP readings (beta: 0.38; B: 3.47; 95%CI: 3.21, 3.73; *P* < 0.001), and higher rise in IOP readings by medical mydriasis (beta: 0.07; B: 0.88; 95%CI: 0.54, 1.22; *P* < 0.001) (Table 3). If the parameter of region of habitation and serum bilirubin concentration were separately dropped from the analysis, the other results remained mostly unchanged. If the parameters of diabetes prevalence (*P* = 0.43), glucose serum concentration (*P* = 0.89), body height (*P* = 0.14), previous cataract surgery (*P* = 0.10), axial length (*P* = 0.18) or prevalence of glaucoma (*P* = 0.11) were added to the model, they were not significantly associated with CCT. If the parameter of diabetes prevalence was kept in the list of independent parameters, its associations with a thicker CCT remained to be statistically significant, if the list additionally contained the parameters of age, sex, region of habitation, ethnicity, level of education, bilirubin serum concentration, corneal refractive power, and anterior chamber angle. If IOP was further added to the model, the parameter of diabetes prevalence lost its significance with CCT (*P* = 0.16). In univariable analysis, a higher diabetes prevalence correlated with a thicker CCT (*P* < 0.001; beta: 0.047).

**Table 3:** Associations (multivariable analysis) between central corneal thickness and ocular parameters in the Ural Eye and Medical Study.

Parameter	Interval	Standardized regression coefficient beta	Non- standardized regression coefficient B	95% confidence interval of B	*P*-Value
Age	Years	−0.09	−0.29	−0.39, −0.20	<0.001
Gender	Women/Men	0.05	3.10	1.18, 5.03	0.002
Region of Habitation	Rural/Urban	0.10	6.83	4.61, 9.05	<0.001
Ethnicity	Non-Russian/Russian	0.04	3.48	1.04, 5.91	0.005
Level of education	Illiteracy/Passing 5^th^ Grade /8th Grade/10th Grade/ 11th Grade/Graduates / Specialized Secondary Education/Post Graduates	0.04	0.97	0.29, 1.66	0.006
Serum concentration of bilirubin	µmol/L	0.05	0.15	0.07, 0.23	<0.001
Corneal refractive power	Dioptres	−0.09	−1.92	−2.50, −1.35	<0.001
Anterior chamber angle	Degree	−0.07	−0.38	−0.52, −0.24	<0.001
Intraocular pressure readings	mmHg	0.07	0.88	0.54, 1.22	<0.001
Medical mydriasis associated rise in intraocular pressure	mmHg	0.38	3.47	3.21, 3.73	<0.001

The mean inter-eye difference in CCT was 8.52 ± 13.9 µm (median: 6.0; 95%CI: 8.16, 8.88) (Fig. 2). A higher inter-eye CCT difference was associated with older age (beta:0.08; B: 0.11; 95%CI: 0.07, 0.15; *P* = 0.01), lower level of education (beta: −0.04; B: −0.34; 95%CI: −0.60, −0.08; *P* < 0.001) and status after cataract surgery (beta: 0.04; B: 2.92; 95%CI: 1.02, 4.83; *P* = 0.003)

## Discussion

In our ethnically mixed population from Russia, CCT (mean: 541.7 ± 33.7 µm) increased with the systemic parameters of younger age, higher level of education and higher serum bilirubin concentration, and with the ocular parameters of lower corneal refractive power, smaller anterior chamber angle, higher IOP readings, and higher rise in IOP readings by medical mydriasis. In addition, CCT was larger in men than women, urban versus rural region of habitation, and in Russians (543.4 ± 31.6 µm) versus non-Russians (539.2 ± 33.9 µm). In that multivariable model, CCT was not associated with body height, previous cataract surgery, axial length or prevalence of glaucoma A higher inter-eye difference in CCT (mean: 8.52 ± 13.9 µm) was correlated with older age, lower level of education and status after cataract surgery.

The mean CCT of 543 µm in Russians was considerably larger than the mean CCT found in Indians (514 µm; Central India and Medical Study; 504 µm, South Indian Chennai Glaucoma Study), Japanese (521 μm, Tajimi Study), indigenous Australians (512 mm) and Afro-Americans (530 mm; Barbados Eye Study), and it is comparable with the mean CCT reported for West-Europeans (537 μm, Rotterdam Study), North Chinese (556 µm, Beijing Eye Study), and Malays (541 μm, Singapore Malay Eye Study) [8, 12–15, 22–24]. Interestingly, the CCT in the non-Russian group in our study population, including Bashkirs, Tartars, Chuvash and other ethnic groups, was significantly thinner (539.2 ± 33.9 µm; *P* < 0.001) than in the Russian group. The findings obtained in our study further support the dependence of CCT on the ethnic background, so that the latter should be taken into account in the diagnosis of glaucoma, if the CCT measurements are not available.

The observation made in our study that CCT was not related with axial length, confirms previous investigations on other ethnic groups [11, 14, 23]. It supports the notion that myopic axial elongation occurs predominantly in the posterior hemisphere of the globe, while the anterior ocular segment including the cornea and its diameter and thickness is not affected by the process of axial elongation [25]. In our study population thicker CCT was associated with a lower corneal refractive power (i.e., a greater radius of corneal curvature or a flatter cornea). The correlation remained to be statistically significant, if eyes with a corneal refractive power of more than 45 dioptres were excluded, so that the relationship was not due to the inclusion of eyes with a keratoconus. The association between a larger thickness and more pronounced flatness of the cornea is of clinical interest since both parameters, a thicker cornea and a flatter cornea, lead to an underestimation of the true IOP [26, 27].

As also noted in numerous previous studies, a thicker CCT was associated with higher IOP readings [1, 2, 8–10, 13–15, 22–24]. In addition to this dependence of the IOP readings on CCT, the IOP measurements depend on the corneal flatness: the flatter the cornea is, the easier it is to applanate the cornea [26, 27].

Interestingly, CCT was not related with the prevalence of glaucoma as a whole or with the prevalence of open-angle glaucoma or angle-closure glaucoma. It is partially in contrast to the results of the Ocular Hypertension Treatment Study in which a thinner cornea at baseline was associated with a higher risk of developing glaucomatous optic nerve damage [7]. In agreement with other studies, such as the Liwan Study, it supports the notion that CCT is not a risk factor for glaucomatous optic neuropathy if the dependence of the IOP measurements on CCT have been taken into account during the diagnosis of glaucomatous optic neuropathy [28–31]. It fits with the results of a histomorphometric study that CCT and thickness of the lamina cribrosa were not significantly correlated with each other [32].

The prevalence of diabetes or the glucose serum concentration did not correlate with CCT in our study population. This observation does not agree with the finding made in the Singapore Malay Eye Study, in which CCT, as measured by ultrasound pachymetry and after controlling for age and gender, was significantly higher in individuals with diabetes than in those without diabetes (*P* < 0.001), and in which higher CCT was associated with higher serum glucose concentration (*P* = 0.02) and higher HbA1c value (*P* < 0.001) [33]. As in our study, other investigations, such as the Iranian Yazd Eye Study, the Korean Namil Study and the South Indian Sankara Nethralaya Diabetic Retinopathy also did not find an association between CCT and diabetes [34–36].

In our study population, CCT was statistically independent of additional other major non-ophthalmological disease such as arterial hypertension, chronic obstructive pulmonary disease, asthma, chronic kidney disease, hepatic disorders associated with an increase in the serum concentration of transaminases, hearing loss, depression and anxiety. Our investigations extends the findings obtained in previous studies on missing relationships between CCT and non-ophthalmological parameters.

Interestingly, CCT was significantly higher in the left eyes than in the right eyes (543.1 ± 33.7 µm versus 541.7 ± 33.7) µm in our study. The reason for this inter-ocular difference has remained unclear. If it was an artefact, its effect on the study results may have been small, since the data of a randomly chosen eye per individual was taken for further statistical analysis.

When the results of our study are discussed, its limitations should be taken into account. First, the value of an epidemiological investigation is profoundly connected with the rate of participation and how much the study area and study population are representative for the region and population it aimed at. In the Ural Eye and Medical Study, the participation rate was 80.5% out of the eligible population of 7328 individuals, and the present study included 98.2% of these participants. There was however a significant difference in age and sex between the participants and non-participants. Second, the study areas were typical for Southern Russia with respect to its demography, geography and climate. The fraction of Russians in our study population and in the study region was lower than in North-Western Russia and Central Russia. To address that issue, we included the parameter of ethnic background into the multivariable analysis and found a significant difference in CCT between Russians and non-Russians. In a study including only Russians this finding would not have been detected. Third, we used Scheimflug imaging for the CCT measurements, while other investigations applied optical low-coherence reflectometer pachymetry or sonographic pachymetry for the determination of CCT. Studies showed that the CCT data obtained by Scheimflug imaging could be compared with those measured by optical low-coherence reflectometer pachymetry [37, 38]. Even if there was a systemic difference in the CCT measurements between studies due to differences in the techniques applied, such a difference might not have affected the associations between CCT and other ocular and general parameters as examined in the present study. Strengths of the Ural Eye and Medical Study were the relatively large study population and the relatively high number of ocular and systemic disorders and parameters assessed and included into the statistical analysis.

In conclusion, in a typical, ethnically mixed, population from Russia with an age of 40+ years, mean CCT (541.7 ± 33.7 µm) was associated with parameters such as younger age, male sex, Russian ethnicity, and higher educational level. These associations may be taken into account when the dependence of IOP readings on CCT are considered. Glaucoma prevalence was unrelated to CCT.

### Summary

#### What was known before

- Central corneal thickness (CCT) is a clinically important parameter in the diagnosis of glaucoma, since the measurement of intraocular pressure markedly depends on CCT. It has additionally been discussed that a thin cornea may be a structural risk factor for an increased susceptibility for glaucomatous optic nerve damage at a given IOP.

#### What this study adds

- In this ethnically mixed population from Russia with an age of 40+ years, mean CCT (541.7 ± 33.7 µm) was associated with parameters such as younger age, male sex, Russian ethnicity, and higher educational level. These associations may be taken into account when the dependence of IOP readings on CCT are considered.
- Glaucoma prevalence was unrelated to CCT.

## Data Availability

The microdata of the study are available from the corresponding authors upon reasonable request.
